# Molecular Regulation of Post‐Hatching Embryo Development: Implications for Early Pregnancy Losses in Livestock Species

**DOI:** 10.1111/rda.70225

**Published:** 2026-09-30

**Authors:** Alexia Siegmund‐Sabater, Nuria Martínez de los Reyes, Inés Flores‐Borobia, Leopoldo González‐Brusi, Beatriz Galiano‐Cogolludo, Pilar Marigorta, Pablo Bermejo‐Álvarez, Priscila Ramos‐Ibeas

**Affiliations:** ^1^ Departamento de Reproducción Animal INIA, CSIC Madrid Spain

**Keywords:** bovine, embryo, ovine, post‐hatching, pregnancy loss

## Abstract

Pregnancy loss (PL) remains a major limitation to reproductive efficiency in livestock, with a substantial proportion originating during early embryo development, particularly between blastocyst hatching and implantation. This developmental window encompasses complex processes of lineage differentiation and morphogenesis. Accumulating evidence indicates that intrinsic defects in embryonic development, most notably within the epiblast, are key contributors to PL, especially following in vitro embryo production. Mechanistic investigation of this period has historically been limited by reliance on resource‐demanding in vivo approaches. In this review, we summarize current knowledge on the molecular regulation of early embryo development in ungulates and the factors associated with PL during the post‐hatching period. We further synthesize recent advances in molecular technologies, extended embryo culture systems, and three‐dimensional endometrial models that are transforming the study of post‐hatching embryo development in livestock species. Collectively, these advances provide a foundation for refining assisted reproductive technologies and developing predictive strategies to mitigate PL, thereby enhancing reproductive efficiency and sustainability in livestock production systems.

## 1 Introduction

Reproductive efficiency is essential for the sustainability of animal production systems. Successful reproduction depends on a series of tightly regulated biological processes, including gametogenesis, fertilization, embryonic and foetal development, and ultimately parturition. However, pregnancy loss (PL) has a major negative impact on animal production systems. The incidence of PL varies substantially depending on the production system and reproductive management. For example, PL is generally higher in dairy compared to beef cattle, likely reflecting differences in metabolic stress (Reese et al. 2020; Sartori et al. 2025), and also differs according to the reproductive strategy used, including timed artificial insemination (AI) and timed embryo transfer of fresh or frozen in vitro‐produced embryos. Notably, PL is higher following ET compared to AI, and calving rates are reduced after transfer of frozen embryos relative to fresh ET or AI (Crowe, Sánchez, Moore, McDonald, McCabe, et al. 2025). Although failures may occur at any stage, PL during the first weeks of gestation represents a major limitation to reproductive performance and economic viability, reaching up to 50% in cattle (Berg et al. 2022; Crowe, Sánchez, Moore, McDonald, McCabe, et al. 2025; Dunne et al. 2000; Wiltbank et al. 2016) and up to 40% in ewes and pigs (Almeida and Dias 2022; Diskin and Morris 2008; Wilmut et al. 1986). During this period, tightly coordinated events of cell proliferation and lineage specification are required to ensure successful implantation and subsequent foetal development.

Following fertilization, the zygote undergoes cleavage divisions culminating in blastocyst formation between days (D) 6 and 8 in ungulates. Embryonic losses during this first developmental stage are estimated to range between 10% and 28% in cattle (Diskin and Sreenan 1980; Reese et al. 2020; Roche et al. 1981) (Figure 1). Until the blastocyst stage, the embryo remains enclosed within the *zona pellucida*. Hatching from this glycoprotein shell marks the onset of post‐hatching development, a second developmental period characterized by complex lineage specification events and extensive morphogenetic remodelling. In rodents and primates, the hatched blastocyst implants shortly thereafter. In contrast, ungulates undergo a prolonged preimplantation period known as conceptus elongation, during which rapid proliferation of extraembryonic membranes transforms the spherical blastocyst into a filamentous conceptus prior to implantation. Concurrently, gastrulation and somitogenesis occur within a flat embryonic disc (ED) while the conceptus remains free‐floating in the uterine lumen (Pérez‐Gómez, Gonzalez‐Brusi, Bermejo‐Alvarez, et al. 2021). This poorly accessible developmental window, often referred to as the ‘black box’ of development, represents the period most susceptible to PL in livestock (Martínez De Los Reyes et al. 2026). Post‐hatching PL in cattle range between 26% and 34% (Berg et al. 2010; Pérez‐Gómez, Gonzalez‐Brusi, Bermejo‐Alvarez, et al. 2021), with approximately one‐third of viable blastocysts failing to complete conceptus elongation (Santos et al. 2004). In contrast, PL after the first month of gestation accounts for less than 6% (Reese et al. 2020) (Figure 1). Notably, comparable patterns are observed in humans, which also develop a flat ED: only ∼30% of conceptions result in live birth, while ∼60% are lost before or around implantation during the first 3 weeks of development, often without clinical detection (Larsen et al. 2013).

**FIGURE 1:**
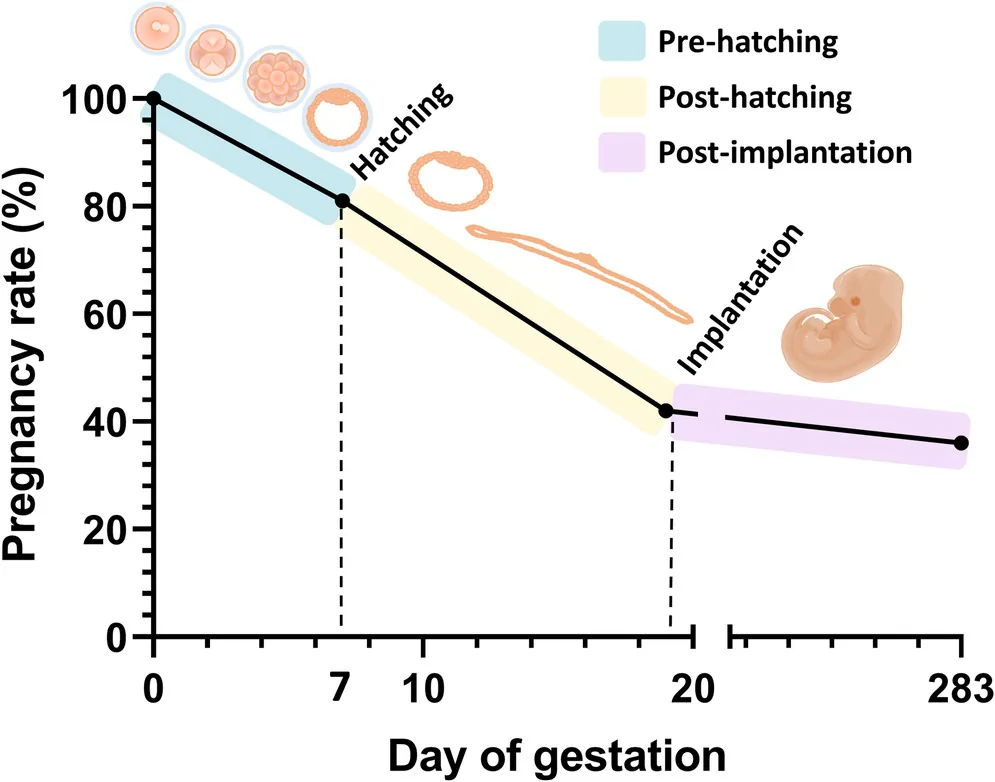
Average pregnancy rate during the pre‐hatching, post‐hatching, and post‐implantation stages in cattle. Representative embryo developmental stages corresponding to each period are shown.

Collectively, these observations underscore the urgent need to elucidate the molecular mechanisms regulating post‐hatching development in ungulates, which are the basis for understanding early PL in livestock. Historically, progress has been constrained by the difficulty of recapitulating post‐hatching development in vitro and by the limited availability of molecular tools for functional studies in non‐rodent species. However, recent advances in extended embryo culture systems, single‐cell transcriptomics, genome editing, and endometrial models now enable mechanistic investigations of early embryo development in livestock. In this review, we discuss the molecular regulation of early embryo development in ungulates and its association with PL, highlighting emerging technologies that are transforming the field.

## 2 Molecular Regulation of Embryo Development in Ungulates

Early mammalian embryogenesis has been extensively characterized in the mouse, which has served as the principal experimental model for several decades. Although the initial stages of embryo development are broadly conserved across mammals, accumulating evidence reveals substantial divergence in gene regulatory networks between rodents and non‐rodent species, particularly ungulates.

During early cleavage stages, embryos rely on maternal transcripts and proteins until embryonic genome activation (EGA), which occurs at the 2‐cell stage in mice and at the 4–8‐cell stage in ungulates and primates (Piliszek and Madeja 2018) (Figure 2). In the mouse, polarization of outer blastomeres at the 8‐cell stage initiates trophectoderm (TE) versus inner cell mass (ICM) specification via Hippo/YAP signalling (Korotkevich et al. 2017; Maître et al. 2016). Activation of *Tead4* in outer blastomeres promotes the expression of TE‐specific genes, including *Cdx2, Elf5*, and *Eomes*, while repressing pluripotency factors such as *Oct4*, *Nanog*, and *Sox2*. This establishes mutually exclusive expression domains of TE and ICM transcription factors prior to blastocyst formation (Niwa et al. 2005; Wicklow et al. 2014).

**FIGURE 2:**
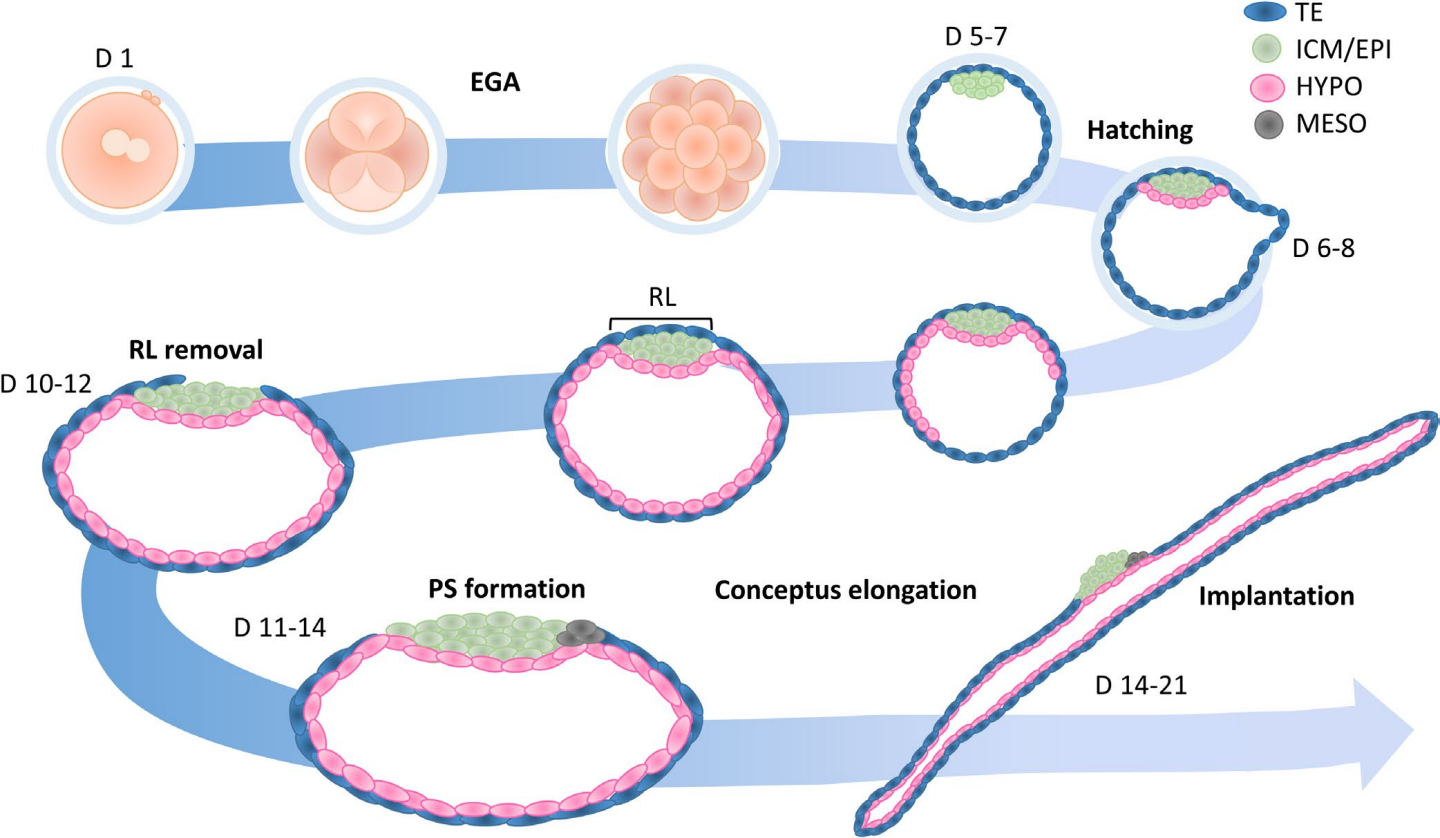
Pre‐implantation embryonic development in ungulate species. Embryonic genome activation (EGA) occurs around the 4‐ to 8‐cell stage. By day (D) 5–7, a blastocyst composed of trophectoderm (TE) and Inner Cell Mass (ICM) cells is formed. Around blastocyst hatching from the *zona pellucida*, the ICM differentiates into the epiblast (EPI) and the hypoblast (HYPO). Hypoblast cells will subsequently migrate along the inner surface of the TE. After formation of the embryonic disc (ED) from the EPI, the polar TE, termed Rauber's layer (RL), is eliminated around D10–12, preceding the emergence of the first mesoderm (MESO) cells and primitive streak (PS) formation during conceptus elongation. In ungulates, all of these events occur prior to implantation.

In contrast, ungulates display substantial differences in the timing and function of these key transcription factors. In cattle, pigs and sheep, CDX2 protein expression is limited prior to blastocyst formation and, in contrast to the mouse, it does not repress OCT4 in TE cells until late blastocyst stages (Berg et al. 2011; Goissis and Cibelli 2014; Kirchhof et al. 2000; Kuijk et al. 2008; Martínez De Los Reyes, Carvajal‐Serna, et al. 2024). Furthermore, *EOMES* and *ELF5* are not expressed in porcine and bovine blastocysts (Ramos‐Ibeas et al. 2019; Wei et al. 2017). *TEAD4*, which is essential for blastocyst formation in mice (Nishioka et al. 2008; Yagi et al. 2007), is dispensable in cattle, rabbits, and humans (Pérez‐Gómez, González‐Brusi, Flores‐Borobia, Galiano‐Cogolludo, et al. 2024; Pérez‐Gómez, González‐Brusi, Muniesa‐Martínez, et al. 2021; Wu et al. 2024). Similarly, *CDX2* deficiency prevents blastocyst formation in mice (Strumpf et al. 2005) but not in bovine embryos (Goissis and Cibelli 2014; Shi et al. 2023). In contrast, *OCT4* ablation impairs blastocyst development in human embryos but not in mice (Fogarty et al. 2017; Stamatiadis et al. 2021), and conflicting results have been reported in bovine embryos (Daigneault et al. 2018; Luo et al. 2022; Simmet et al. 2018).

At the time of blastocyst hatching, the second lineage segregation gives rise to the epiblast, which will form the future foetus, and the hypoblast, which together with the TE form the extraembryonic membranes. In mice, epiblast versus hypoblast specification is driven by mutually exclusive expression of *Nanog* and *Gata6* within the late ICM (Plusa et al. 2008). However, this ‘salt‐and‐pepper’ pattern is not conserved in primates and ungulates, where *GATA6* expression is not initially restricted to the hypoblast and becomes lineage‐specific only at later stages (Blakeley et al. 2015; Kuijk et al. 2008; Martínez de Los Reyes, Flores‐Borobia, et al. 2024; Ramos‐Ibeas et al. 2019).

Despite the morphological similarities, these interspecies differences in the molecular regulation of pre‐hatching development challenge mouse‐centred paradigms of early embryo development.

In contrast to the shared morphology found during pre‐hatching embryo development, post‐hatching development differs markedly across mammalian species. Both rodent and primate embryos implant into the uterus shortly after hatching, whereas ungulates undergo an extended pre‐implantation period known as conceptus elongation. Following blastocyst hatching, the hypoblast proliferates and migrates to cover the inner surface of the mural TE, thereby enclosing the blastocoel cavity. The polar TE, which is in direct contact with the epiblast, forms Rauber's layer (RL) in ungulates. This transient structure undergoes apoptosis prior to elongation, exposing the epiblast to the uterine environment (van Leeuwen et al. 2020). After RL removal, both the mural TE and the hypoblast undergo extensive expansion (Figure 2). This coordinated growth transforms the embryo from spherical to ovoid, tubular and ultimately filamentous stages, reaching up to 20 cm in sheep (Davenport et al. 2023), 30 cm in cattle (Maddox‐Hyttel et al. 2003), and 100 cm in pigs (Geisert et al. 1982) by the time of implantation, which occurs approximately at D14 in pigs (Waclawik et al. 2017), D15 in sheep (Guillomot et al. 2004), and D19 in cattle (Bazer et al. 2009).

In contrast to the mouse embryo, in which the epiblast forms a three‐dimensional cup‐shaped structure where gastrulation takes place, primate and ungulate embryos develop a flat ED. In these species, an increased cell density at the posterior region of the ED precedes the formation of the primitive streak (PS), marking the onset of gastrulation and the establishment of the three germ layers: ectoderm, mesoderm, and endoderm (Guillomot et al. 2004; Kobayashi et al. 2017) (Figure 2). Current understanding of mammalian gastrulation is largely derived from studies in the mouse. However, key structures involved in gastrulation in the mouse, such as the extraembryonic ectoderm (ExE), which is derived from the polar TE and serves as a major source of signalling cues, are absent in non‐rodent species (Bedzhov et al. 2014; Martínez De Los Reyes et al. 2026). In mice, *BMP4* produced by the ExE is essential for PS formation (Winnier et al. 1995). In contrast, in ungulates, the polar TE‐derived RL disappears prior to gastrulation (van Leeuwen et al. 2020), and *BMP4* expression is not detected until the emergence of mesoderm cells at the onset of PS formation (González‐Brusi et al. 2026; Simpson et al. 2024). Consequently, the potential signalling gradients and molecular mechanisms driving gastrulation in ungulates remain poorly defined.

## 3 Factors Associated With PL During Post‐Hatching Development in Livestock

Traditionally, PL during post‐hatching development has been primarily attributed to insufficient conceptus elongation, failures in embryo‐maternal communication, or uterine dysfunction (Sartori et al. 2025; Wiltbank et al. 2016). The timing of conceptus attachment—assessed by rising concentrations of pregnancy‐specific protein B (PSBP) in maternal blood—has also been associated with PL. Cows exhibiting a delayed increase in PSBP, indicative of prolonged post‐hatching development, show a higher likelihood of PL (Crowe, Sánchez, Moore, et al. 2025; Santos et al. 2023).

After entering the uterus at the morula stage, the embryo undergoes blastocyst formation and hatching, followed by conceptus elongation prior to placentation. During this period, the embryo is entirely dependent on uterine secretions collectively termed histotroph. The histotroph is primarily produced by the glandular epithelium and consists of a complex mixture of proteins, lipids, amino acids, growth factors, extracellular vesicles (EVs), and a wide range of bioactive molecules (Forde, McGettigan, et al. 2014; Forde, Simintiras, et al. 2014; Nakamura et al. 2019; Simintiras et al. 2019). Its essential role in post‐hatching development is supported by functional evidence demonstrating that the absence of endometrial glands in sheep results in failure of conceptus elongation (Gray et al. 2001). Importantly, histotroph composition changes dynamically during early pregnancy probably matching embryonic needs (Mathew et al. 2022). Consequently, alterations in its composition can compromise post‐hatching development and predispose to PL (Figure 3).

**FIGURE 3:**
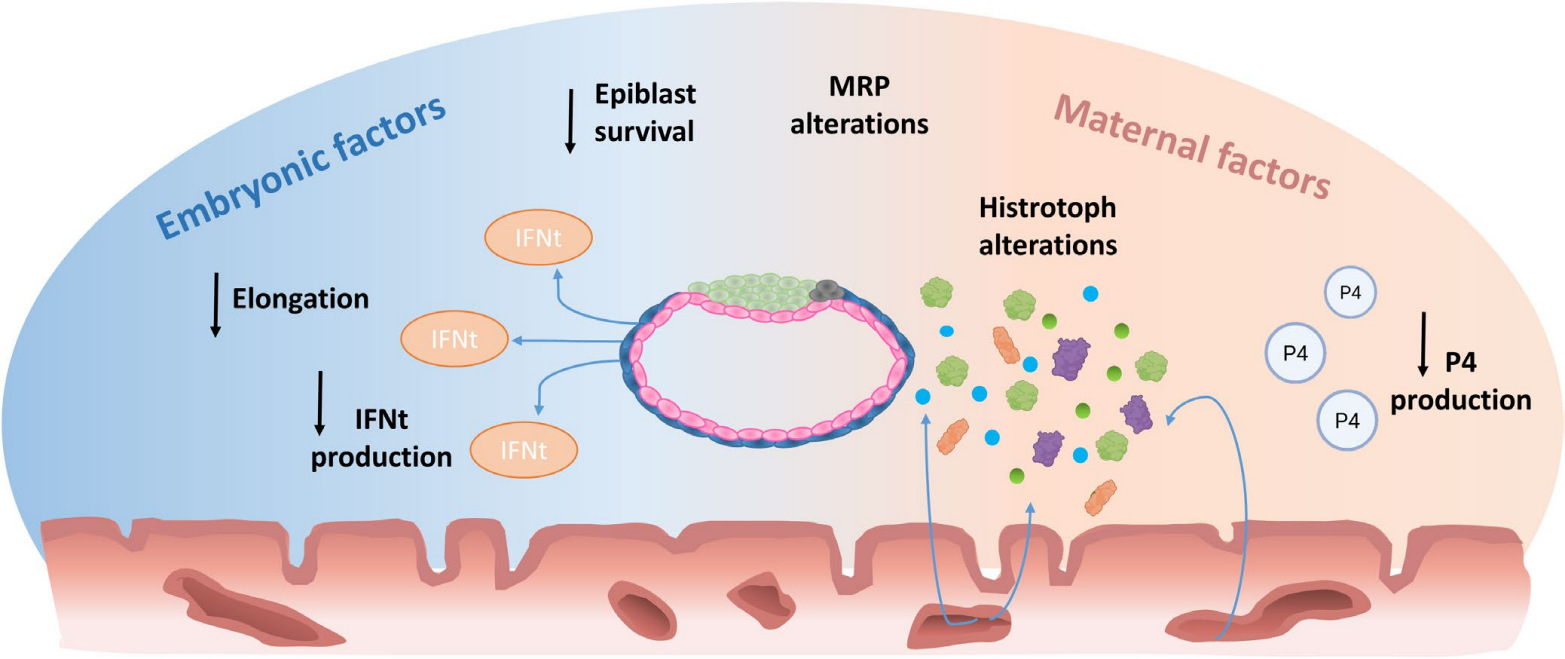
Schematic representation of proposed embryonic and maternal factors involved in post‐hatching pregnancy loss in ruminants. IFNt, Interferon tau; MRP, maternal recognition of pregnancy; P4, progesterone.

At the end of a normal estrous cycle, pulsatile secretion of prostaglandin F2α (PGF2α) induces regression of the corpus luteum (CL) (Wathes and Lamming 1995). In ruminants, the presence of an embryo leads to transient suppression of endometrial PGF2α synthesis induced by TE‐derived interferon‐tau (IFNT), thereby maintaining CL function and continued progesterone (P4) production. This process, termed maternal recognition of pregnancy (MRP), is essential for pregnancy establishment (Brooks et al. 2014; Mathew et al. 2022; Rowson and Moor 1967). Some studies suggest that the magnitude of the endometrial response to the embryo is influenced by conceptus size (Sánchez et al. 2019) or by IFNT concentration (Talukder et al. 2023). In addition, endometrial responsiveness to IFNT varies among individuals and has been reported to correlate with subsequent fertility in dairy heifers (Madureira et al. 2024). However, recent evidence challenges the prevailing MRP model in ruminants. Pregnancies have been reported in cattle following transfer of *IFNT* knockout (KO) embryos (Wolf et al. 2026), and lambs have been born after natural mating from sheep KO for *IFNAR2*, encoding interferon alpha and beta receptor subunit 2 (Davies et al. 2024). These studies are currently under review and reported in an abstract, respectively. Thus, further functional studies are required to determine whether alternative or redundant mechanisms for pregnancy recognition exist.

Classical studies estimate that approximately one‐third to one‐half of PL is attributable to maternal factors, whereas the remaining proportion results from embryo‐related causes (Domingues et al. 2023; Ealy et al. 2019). Among the latter, embryo quality and origin‐i.e., in vitro‐produced (IVP) vs. in vivo‐derived‐are major determinants of PL (Ealy et al. 2019; McMillan et al. 1997; Sartori et al. 2025). In cattle, the number of commercially produced IVP embryos has increased markedly, exceeding 2 million worldwide in 2024, compared with fewer than 300,000 in vivo‐derived embryos (Viana 2025). Despite this expansion, the developmental competence of IVP embryos remains suboptimal. PL following AI are estimated at 45%–65% in lactating dairy cattle and 10%–30% in beef cattle (Ealy et al. 2019; Gatea et al. 2018; Lucy 2001; Pohler et al. 2015; Santos et al. 2004; Wiltbank et al. 2016). In contrast, pregnancy failure rates in recipients of IVP embryos range from 59% to 85%, with approximately 40% of cows receiving an IVP blastocyst no longer pregnant by D18‐21 of gestation (Block and Hansen 2007; Ealy et al. 2019; McMillan et al. 1997; Reichenbach et al. 1992). These findings point to profound alterations in post‐hatching development associated with IVP.

At the level of the extraembryonic membranes, IVP conceptuses exhibit delayed or inefficient early elongation, with partial compensatory growth in surviving conceptuses (Barnwell et al. 2015; Bertolini, Beam, et al. 2002; Lonergan et al. 2007). Altered TE function has been inferred from differences in endometrial transcriptomic responses to IVP compared to in vivo‐derived conceptuses (Mansouri‐Attia et al. 2009; Mathew et al. 2019). Although IFNT has been proposed as a mediator of these effects, reports on IFNT mRNA and protein abundance in IVP versus in vivo embryos are inconsistent, and its contribution to altered endometrial signalling remains unclear (Arnold et al. 2006; Bertolini, Beam, et al. 2002; Kubisch et al. 2001; Stojkovic et al. 1995). Although comparatively understudied, IVP pregnancies have also been associated with impaired development of the yolk sac, a hypoblast‐derived extraembryonic structure that mediates nutrient and gas exchange with the histotroph (Freyer and Renfree 2009; Jollie 1990; Shi et al. 1985). These abnormalities are characterized by defective villous architecture, reduced vascularization, and transcriptional alterations (Alberto et al. 2013; Mess et al. 2017).

Although developmental defects in extraembryonic lineages may occur, the epiblast appears to be the lineage most susceptible to environmental insults occurring during post‐hatching development (Figure 3). A substantial proportion of IVP‐derived conceptuses (23%–65%) exhibit normal TE and hypoblast development but lack an ED, resulting in anembryonic pregnancies incapable of foetal development (Alexopoulos et al. 2008; Bertolini, Beam, et al. 2002; Bertolini, Mason, et al. 2002; Block et al. 2007; Desmet et al. 2020; Fischer‐Brown et al. 2004; Ideta et al. 2007; Loureiro et al. 2011) (reviewed in Ealy et al. 2019; Pérez‐Gómez, Gonzalez‐Brusi, Bermejo‐Alvarez, et al. 2021). This lineage‐specific susceptibility is evident from the blastocyst stage, when IVP embryos show reduced ICM cell numbers (Gjorret et al. 2003; Iwasaki et al. 1990; Knijn et al. 2003; Pomar et al. 2005). Consistently, IVP‐derived conceptuses frequently display smaller EDs than in vivo‐derived counterparts (Bertolini, Beam, et al. 2002), and pregnancy retention rates are dramatically reduced following transfer of IVP conceptuses lacking a detectable ED compared with those with visible EDs (62.5% vs. 6.7%, respectively) (Bertolini, Mason, et al. 2002). Comparable phenotypes have been described in ovine and bovine embryos generated by somatic cell nuclear transfer (SCNT), a technology associated with widespread developmental perturbations (Chavatte‐Palmer et al. 2012; Hall et al. 2013). In SCNT embryos, the incidence of ED absence ranges from 20% to 58% (Alexopoulos et al. 2008; Degrelle et al. 2012; Ideta et al. 2007; Rodríguez‐Alvarez et al. 2010; Smith et al. 2010; Tveden‐Nyborg et al. 2005), and transcriptional alterations are disproportionately more evident in the epiblast relative to extraembryonic lineages (Alexopoulos et al. 2008; Degrelle et al. 2012; Hirasawa et al. 2013; Oda et al. 2010).

This phenomenon has often been overlooked, as many studies assessing embryo quality during elongation have relied primarily on conceptus length without evaluating ED formation (Barnwell et al. 2016; Kasimanickam et al. 2021; O'Hara et al. 2014; Sánchez et al. 2019). Importantly, early pregnancy detection methods in cattle, such as assessment of interferon‐stimulated genes in peripheral granulocytes (Kizaki et al. 2013) and detection of pregnancy associated glycoproteins (PAGs) in milk or blood (Filho et al. 2020; Roberts et al. 2015), rely exclusively on signals derived from the TE and therefore cannot confirm proper ED formation or foetal development. Consequently, epiblast‐specific developmental failures often escape pregnancy detection and ultimately manifest as late PL (Fischer‐Brown et al. 2004). Moreover, because pregnancy diagnosis is typically performed around D30, most early PL remain undetected (Sartori et al. 2025).

Collectively, these observations indicate that although PL during post‐hatching development may result from an inadequate maternal environment and/or impaired embryo‐maternal communication, intrinsic defects in embryonic development are major contributors to post‐hatching PL. In this sense, in vitro embryo production can impair proper lineage differentiation and morphogenetic processes during post‐hatching development, with a particularly pronounced impact on the epiblast.

## 4 Emerging Technologies to Investigate the Molecular Regulation of Post‐Hatching Development and Associated PL

Investigation of post‐hatching development has historically been constrained by the reliance on labour‐intensive and resource‐demanding in vivo approaches, including embryo transfer followed by posterior recovery. Fortunately, the emergence of extended embryo culture systems, three‐dimensional (3D) endometrial culture platforms, and advanced molecular technologies, such as single‐cell transcriptomics and genome editing, has begun to provide new insights into the regulation of post‐hatching development and its association with PL (Figure 4).

**FIGURE 4:**
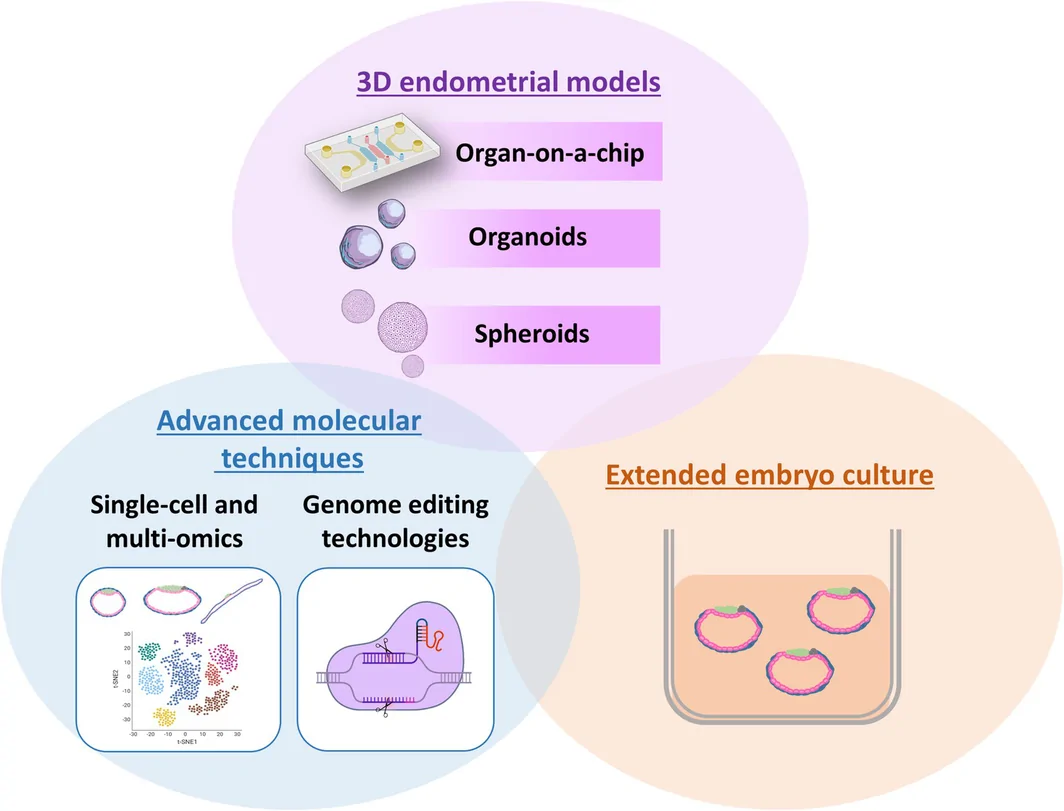
Emerging technologies to investigate the molecular regulation of post‐hatching development in livestock. The integration of advanced molecular techniques, extended embryo culture platforms, and three‐dimensional (3D) endometrial models has the potential to unravel the mechanisms driving post‐hatching development, as well as the underlying causes of pregnancy loss during this period.

### 4.1 Modelling Embryo‐Maternal Communication in Vitro During Post‐Hatching Development

The application of 3D endometrial culture systems to livestock species offers a scalable and high‐throughput strategy to model embryo‐maternal crosstalk during conceptus elongation, with improved physiological relevance compared to conventional 2D cultures. Microfluidic and organ‐on‐a‐chip technologies enable the controlled co‐culture of endometrial epithelial and stromal cells, allowing manipulation of hormonal and metabolic conditions (De Bem et al. 2021). Spheroids represent relatively simple 3D cellular aggregates and have been used in bovine to assess endometrial responses to key pregnancy‐associated molecules such as IFNT and P4 (Rahman et al. 2020). Organoids, by contrast, are derived from tissue‐specific progenitor cells, are self‐organizing, and require an extracellular matrix to form (Gunti et al. 2021). Endometrial organoids (EOs), initially established in mouse and human, recapitulate key features of the in vivo endometrium, support long‐term expansion and hormonal responsiveness to model different stages of the estrous cycle, and are mainly composed of glandular cells, responsible for histotroph secretion in ungulates (Boretto et al. 2017; Fitzgerald et al. 2019; Turco et al. 2017).

In livestock, porcine EOs have been developed as a platform to investigate embryo‐maternal crosstalk during conceptus elongation; however, these EOs were derived from uteri at the metaestrous stage, and evidence supporting embryo viability and conceptus elongation within this in vitro system has not yet been demonstrated (Saadeldin et al. 2024). Similarly, EOs from bovine uteri at the estrous or mid‐late luteal phase, which are responsive to IFNT and P4, have been established and characterized (Devkota et al. 2026; Edge et al. 2025). Nevertheless, blastocysts cultured with intra‐organoid fluid did not exhibit improved developmental outcomes (Devkota et al. 2026). Derivation of EOs from pregnant uteri during the conceptus elongation phase would more accurately reflect the physiological uterine environment encountered by the embryo. Integration of such stage‐matched EO systems with extended embryo culture platforms has the potential to extend the current window of development in vitro. Importantly, this combined approach would provide an experimentally tractable framework to interrogate embryo–maternal signalling mechanisms while reducing dependence on in vivo experimentation.

### 4.2 Investigating Post‐Hatching Development in Vitro Through Advanced Molecular Technologies and Extended Embryo Culture

Recent advances in molecular technologies allow investigation of post‐hatching development at an unprecedented resolution. Single‐cell and multi‐omics approaches currently provide high‐resolution characterization of the developmental timing and molecular mechanisms governing cell fate specification. In livestock species, single‐cell transcriptomic studies have begun to define lineage segregation and pluripotency transitions during conceptus elongation. In pigs, analyses have focused on lineage dynamics and epiblast pluripotency states during early elongation up to E11 spherical/ovoid embryos (Dufour et al. 2024, 2026; Ramos‐Ibeas et al. 2019), as well as on gastrulation up to elongated stages (E11.5 to E15) (Simpson et al. 2024). In cattle, embryos spanning spherical (E12) to elongated (E18) stages have been profiled, with particular emphasis on extraembryonic membranes (Scatolin et al. 2024). In sheep, recent studies have characterized pluripotency transitions up to elongated stages (E14) (Zhang et al. 2025), as well as conceptus elongation, symmetry breaking and gastrulation between E11 and 13.5 (González‐Brusi et al. 2026). Additional work has integrated single‐cell profiling of conceptuses and their corresponding endometrium at pre‐ and peri‐implantation stages (E9 to 19), providing insight into embryo‐maternal signalling interactions (Jia et al. 2023). Importantly, all of these datasets have been generated exclusively from in vivo‐derived embryos.

Conventional embryo culture systems support in vitro development only up to blastocyst hatching, precluding experimental manipulation during later stages. Thus, functional interrogation of post‐hatching development has remained limited, largely due to reliance on embryo transfer followed by conceptus recovery (Alexopoulos et al. 2008; Clemente et al. 2009; Diskin and Morris 2008; van Leeuwen et al. 2014). To overcome this limitation and to support in vitro development of the epiblast, we developed an extended culture system based on N2B27 medium (Ramos‐Ibeas et al. 2022, 2020). N2B27 is a chemically defined medium composed of Neurobasal and DMEM/F12 supplemented with N2 and B27, widely used for embryonic stem cell derivation and maintenance (Nichols and Ying 2006). N2B27 contains amino acids, lipids, vitamins, hormones, and growth factors not present in earlier post‐hatching culture systems, in which the epiblast failed to develop (Alexopoulos et al. 2005; Brandão et al. 2004; Vajta et al. 2004). Using this system, bovine blastocysts develop beyond hatching, achieving complete hypoblast migration and epiblast survival, resulting in formation of an early ED by D12 to 15 (Ramos‐Ibeas et al. 2020, 2023). Building on these findings, the system was adapted for ovine embryos, where further optimization through supplementation with activin A and a ROCK inhibitor supported ED development up to early gastrulation by D14, a key milestone in development (Ramos‐Ibeas et al. 2022). Similarly, other authors have recently reported extended embryo culture media based on uterine composition, diverse growth factors, and B27 supplement—the most successful additive—supporting bovine embryo survival up to D12, and proper lineages development of bovine embryos up to D10 (Isaac et al. 2024; Isaac and Pfeffer 2021).

This in vitro platform represents a substantial advance, as it enables direct assessment of embryo developmental competence during the post‐hatching period without reliance on experimental animals. Traditionally, embryo quality assessment has relied either on the expression of selected molecular markers, often with limited predictive value and low reproducibility (Corcoran et al. 2006; Cánepa et al. 2014; Fujii et al. 2024; Guzeloglu et al. 2026; Rabaglino and Hansen 2024; Suwik et al. 2021), or on embryo transfer experiments, which require large numbers of animals, entail high costs, and are subject to significant biological variability. Extended embryo culture offers a powerful alternative for improving in vitro embryo production protocols and associated technologies, such as embryo vitrification, by allowing direct visualization and quantitative assessment of post‐hatching developmental progression entirely in vitro. Key measurable parameters include embryo survival and growth, hypoblast migration along the inner TE surface, epiblast survival and proliferation, and ED formation.

This system has been used to assess embryo viability in very diverse settings. For instance, we have used it to evaluate the developmental competence of heterologous ibex–goat blastocysts generated from domestic goat oocytes fertilized using different protocols with Iberian ibex sperm, an endemic species from Spain (Martínez de Los Reyes, Carvajal‐Serna, et al. 2024). In a separate application, the platform was used to assess the viability of bovine blastocysts produced following automatic zygote denudation using a microfluidics‐based device. Extended culture revealed proper lineage development and post‐hatching competence, thereby validating the approach (Guerrero‐Sánchez et al. 2025). Independent studies have similarly employed N2B27‐based post‐hatching culture to examine the effects of supplementation with bioactive molecules, including interleukin‐6, interleukin‐11, and leukaemia inhibitory factor (LIF), on vitrified bovine blastocysts after thawing (Oliver et al. 2025, 2024), as well on ED development during post‐hatching development (Oliver et al. 2026). In addition, bovine blastocyst‐like structures derived from stem cells have been cultured in parallel with IVP blastocysts for over 2 weeks, demonstrating sustained growth (Pinzón‐Arteaga et al. 2023).

Beyond embryo quality assessment, post‐hatching in vitro culture allows us to investigate through targeted addition or removal experiments, the roles of hormones, growth factors, metabolites, and signalling pathways whose dysregulation during post‐hatching development may contribute to PL. For instance, pharmacological inhibition of transforming growth factor beta (TGFβ) signalling demonstrated its essential role in epiblast and hypoblast development between D6 and 14 in sheep (Galiano‐Cogolludo et al. 2023). Such functional analyses were previously restricted to pre‐hatching stages, the endpoint of conventional culture systems. Indeed, using conventional embryo culture, we demonstrated that MEK signalling is required for hypoblast specification at the blastocyst stage. Extended culture further revealed its requirement for subsequent hypoblast proliferation and migration between D7‐8 and 12 in ovine embryos (Martínez de Los Reyes, Flores‐Borobia, et al. 2024).

Using this system, we also investigated the role of arachidonic acid (AA) during bovine post‐hatching development. Among the critical lipids present in the histotroph, AA serves as a precursor for prostaglandins synthesis, which play key roles during early embryogenesis (Dorniak et al. 2011; Lewis et al. 1982; Menezo et al. 1982). Whereas pre‐hatching embryos were unresponsive to AA supplementation, elevated AA concentrations significantly impaired survival and growth of post‐hatching embryos (González‐Brusi et al. 2023). Consistent with these observations, we examined the role of peroxisome proliferator‐activated receptor gamma (PPARG), a mediator of lipid and prostaglandin synthesis proposed to be essential for conceptus elongation (Brooks et al. 2015; Cammas et al. 2006; Ribeiro et al. 2016). By combining CRISPR‐mediated gene ablation with extended embryo culture, we demonstrated that embryonic *PPARG* is dispensable for post‐hatching development, a finding subsequently validated in vivo following embryo transfer and recovery (Pérez‐Gómez, González‐Brusi, Flores‐Borobia, Martínez De Los Reyes, et al. 2024).

The platform has also enabled functional analysis of endocrine factors, including melatonin, a hormone central to reproductive seasonality in sheep. While melatonin has been extensively studied during oocyte maturation, fertilization, and pre‐hatching development, extension of these analyses to post‐hatching stages up to D12 revealed no beneficial effects of melatonin supplementation on developmental rates or lineage development. Instead, high concentrations impaired epiblast survival (Carvajal‐Serna et al. 2025).

Combining genome editing technologies with extended embryo culture allows precise functional dissection of the molecular determinants of post‐hatching development. Using this approach, we demonstrated that *TEAD4*, an essential transcription factor for TE specification in mice (Nishioka et al. 2008; Yagi et al. 2007), is dispensable for bovine TE development during both blastocyst formation and post‐hatching stages (Pérez‐Gómez, González‐Brusi, Flores‐Borobia, Galiano‐Cogolludo, et al. 2024; Pérez‐Gómez, González‐Brusi, Muniesa‐Martínez, et al. 2021). Similarly, CRISPR‐mediated knockout of the TE‐associated transcription factor *GATA3* in ovine embryos did not affect TE development but impaired epiblast development during post‐hatching stages (Martínez de Los Reyes et al. 2025).

Finally, this strategy has provided mechanistic insights into early embryonic lethality associated with deleterious haplotypes in cattle. Although several haplotypes have been linked to PL, the precise developmental stage at which arrest occurs remained unknown, despite its importance for reproductive management. We investigated Holstein haplotype 5 (HH5), which involves a mutation in the transcription factor B1 mitochondrial (*TFB1M*) gene and is associated with PL before D60 (Cooper et al. 2014). Using a combination of CRISPR‐mediated gene ablation and extended embryo culture, we determined that *TFB1M*‐deficient embryos develop normally until D12 in vitro but arrest by E14 in vivo, failing to initiate elongation (Pérez‐Gómez, Hamze, et al. 2024). Similarly, we modelled Holstein haplotype 3 (HH3), which involves loss of Structural Maintenance of Chromosome 2 (*SMC2*) gene. Using an exclusively in vitro approach, we demonstrated that *SMC2*‐deficient embryos arrest by D12 without the need for embryo transfers (Pérez‐Gómez, Flores‐Borobia, Hamze, Galiano‐Cogolludo, et al. 2024).

Together, these studies establish the combination of advanced molecular technologies and extended embryo culture as a robust platform for mechanistic investigation of post‐hatching development and PL, enabling functional analyses that were previously inaccessible in livestock species.

## 5 Conclusions and Future Directions

Post‐hatching development represents a critical yet underexplored period in livestock species, during which a substantial proportion of PL originate. Beyond failures in maternal recognition of pregnancy or uterine dysfunction, accumulating evidence indicates that intrinsic defects in embryonic development are major determinants of post‐hatching PL. In vitro embryo production is consistently associated with developmental alterations, with the epiblast displaying a disproportionate vulnerability compared with extraembryonic tissues. This is exemplified as evidenced by the high incidence of IVP‐derived elongated conceptuses lacking an ED. However, the detection of PL associated with this phenotype remains challenging, as current early pregnancy detection methods in cattle rely exclusively on signals derived from the TE. Consequently, detectable early PL likely represent only ‘the tip of the iceberg’, underestimating the true prevalence of embryonic failure.

Despite the absence of major breakthroughs to reduce PL incidence in livestock, significant progress has been achieved in recent years in elucidating the molecular mechanisms governing early embryo development in livestock species. Looking forward, the ongoing revolution in single‐cell omics is expected to further refine our understanding of early embryo development in ungulates. In parallel, the development of extended embryo culture systems, together with genome editing technologies and 3D endometrial models, is transforming our capacity to interrogate post‐hatching development and embryo‐maternal communication in a controlled and mechanistic manner (Figure 4). Beyond advancing fundamental knowledge, these approaches provide a foundation for the development of predictive tools to identify pregnancies at risk of failure and animals predisposed to PL prior to breeding, as well as for the optimisation of assisted reproductive technologies. Ultimately, such advances hold promise for improving reproductive efficiency and the sustainability of livestock production systems.

## Author Contributions

P.R.‐I. conceived the review. A.S.‐S. prepared the figures. A.S.‐S. and P.R.‐I. contributed to literature analysis and manuscript writing. All authors contributed substantially to the development of the concepts discussed in the review, critically revised the manuscript, and approved the final submitted version.

## Funding

This work has been funded by the project PID2024‐155682NB‐I00 from the Spanish Ministry of Science and Innovation.

## Conflicts of Interest

The authors declare no conflicts of interest.

## Data Availability

Data sharing not applicable to this article as no datasets were generated or analysed during the current study.
